# Evaluating the impact of ionic and nano silver on the reproductive dynamics of *Macrogyrodactylus congolensis*: host-dependent and independent effects

**DOI:** 10.1007/s00436-025-08566-1

**Published:** 2025-10-16

**Authors:** Lutfiyya Latief, Tarryn L. Botha, Annemariè Avenant Oldewage

**Affiliations:** Department of Zoology, University of Johannesburg, Auckland Park, Johannesburg, South Africa

**Keywords:** Aquaculture, Monogenea control, Parasite microhabitat, Parasite control, Tegument ultrastructure, Life below water

## Abstract

Ectoparasitic infections pose significant challenges in aquaculture, often leading to high fish morbidity and mortality. *Macrogyrodactylus congolensis*, a viviparous monogenean parasite infecting *Clarias gariepinus*, is of particular concern due to its rapid reproduction and transmission rates. This study investigates the effects of ionic silver (AgNO_3_) and engineered nano-silver (nAg) on the reproductive dynamics and spatial distribution of *M. congolensis*, both on and off its host. Parasite reproduction off the host was assessed under unexposed then varying concentrations of AgNO_3_ (0.1–100 µg/L) and nAg (0.1–100 mg/L), while host-parasite interactions were conducted using the LC_10_ of the parasite for AgNO_3_ and nAg. Results indicate that silver-based treatments exhibit concentration-dependent inhibitory effects on parasite reproduction. At lower concentrations (≤ 2 µg/L AgNO₃ and ≤ 1 mg/L nAg), minimal effects were observed. In contrast, higher concentrations (≥ 50 µg/L AgNO_3_ and ≥ 20 mg/L nAg) significantly suppressed reproduction. Parasite mortality increased with silver exposure, particularly in off-host conditions. Spatial distribution heatmaps illustrated that *M. congolensis* primarily occupied the host’s head region, i.e. preference for microhabitats that support survival and transmission. Additionally, scanning electron microscopy (SEM) analysis of parasites exposed to both forms of Ag showed tegument disruption. The host integument showed minimal punctures when exposed to AgNO_3_ and no damage in the control and exposure to nAg. While AgNO_3_ and nAg show potential as antiparasitic agents in aquaculture, their broader ecological and physiological impacts on host fish and the environment require further investigation. The study highlights the need for optimised dosing strategies to balance efficacy and environmental and host safety, offering valuable insights into developing alternative parasite management approaches for aquaculture.

## Introduction

Freshwater fishes face numerous challenges in aquaculture environments, where high stocking densities and compromised water quality create ideal conditions for parasite infections. Parasites often display specificity in their distribution within/on individual hosts, selecting microhabitats that maximise infection success, growth, reproduction, and transmission (Sukhdeo and Sukhdeo 1994; Poulin 2005; Gobbin et al. 2021; Twumasi et al. 2022). The distribution of parasites on a host is influenced by factors such as resource availability, competition, host immune defences, and environmental parameters like temperature and pH (Holmes 1973; Jensen and Johnsen 1992; Friesen et al. 2018; Zolovs et al. 2018). For example, some hard-bodied ticks (*Ixodes* spp.) attach to the heads of avian hosts, as shorter feathers facilitate longer feeding durations due to minimal host grooming, which improves survival and reproductive success of the parasites (Fracasso et al. 2019).

Variations between individuals in microhabitat selection, driven by environmental factors or competition, highlight the adaptive strategies parasites employ to thrive within or on their hosts (Holmes 1973; Poulin 2007). Monogeneans have gained attention for their rapid transmission rates, which often result in high infection levels and mortality in fish populations, particularly in aquaculture facilities where fish are continuously in close proximity to one another (Kearns 1994; Bakke et al. 2007). This group includes a diverse range of species, but species in the genus *Gyrodactylus* are particularly well studied due to their impact on commercially important fish, like salmonids which were almost wiped out in Norway (Cable and Harris 2002; Johnsen 1978; Tanum 1983; Bauer 1987; Appleby et al. 1997). Fatal infections of the lungfish, *Polypterus senegalus* (Cuvier, 1829), by *Macrogyrodactylus polypteri* (Malmberg, 1957) in aquaria (Khalil 1964) have also been reported. Species of monogeneans exhibit diverse microhabitat preferences that reflect complex host-parasite dynamics formed by ecological and evolutionary pressures. For example, *Macrogyrodactylus clarii* favours the inner hemibranchs on *C. gariepinus* and specifically the dorsal or middle gill segments, likely due to reduced competition and optimal resource availability (Mashaly et al. 2019), whereas a congeneric species, *Macrogyrodactylus congolensis*, occurs exclusively on the skin of the same species.

*Macrogyrodactylus congolensis* is of concern for aquaculture systems due to its high reproductive capability and the ability to cause significant harm to its host (Paperna 1996; Maduenyane et al. 2022a, b). This parasite damages the host’s skin through both attachment and feeding, causing lesions that can become infected with secondary bacterial infections that further weaken the host’s immune system (Kotob et al. 2016). Several studies have confirmed that environmental factors, particularly pollutants, can significantly influence parasite-host interactions in fish. For example, heavy metals can impact host immunity, making fish more susceptible to parasitic infections (Lafferty and Kuris 1999; Sures et al. 2017). Conversely, some pollutants have been shown to have direct toxic effects on parasites (Gilbert and Avenant-Oldewage 2017; Sures et al. 2017; Cuco et al. 2020; Gilbert et al. 2022; Latief et al. 2023; Pretorius et al. 2025), suggesting their potential in parasite control. However, recently, silver, both in ionic form and as nanomaterials, has long been recognised for its antimicrobial properties, which have led to its use in medical applications for wound healing and water treatment (Shobana et al. 2021).

Despite the growing interest in using ionic silver and nAg for controlling/reducing pathogens, there is limited information on their effects on metazoan parasites. Rehman et al. (2019) investigated biologically synthesised nAg on the amphistome, *Gigantocotyle explanatum*, an oviparious trematode, showing that exposure impaired parasite survival and induced oxidative stress, DNA damage, and severe tegument disruption. Exposure to nAg caused tegument swelling and membrane disruption on an oviparous monogenean, *Cichlidogyrus* spp. (Dactylogyridae) (Pimentel-Acosta et al. 2019), leading to reduced survival and reproduction. The study also reported on the inhibition of egg development in these parasites, highlighting the effect of silver nanoparticles on parasite reproduction, which is particularly relevant given that egg stages are often difficult to eliminate with commonly used anti-parasitic treatments in aquaculture. Later, Pimentel-Acosta et al. (2020) demonstrated that nAgs also induce DNA damage and cell death in *Cichlidogyrus* spp. More recently, dos Santos et al. (2024) reported a dose-dependent effect of nAg against another oviparous monogenean, *Dactylogyrus minutus* (Dactylogyridae) in koi carp, with up to 87% efficacy in 300 min of exposure, although complete elimination of these parasites was not observed. However, the manner in which silver and nAg impact the reproductive behaviour and population dynamics of viviparous monogeneans remains unclear. *Macrogyrodactylus congolensis* (Gyrodactylidae) exhibits complex viviparity and progenesis, and is an ideal model to address this gap as these parasites are highly fecund (Cable and Harris 2002).

The present study aimed to evaluate the effects of AgNO_3_ and nAg on the reproductive behaviour, fecundity, and spatial distribution of *M. congolensis* on its fish host, *C. gariepinus*. The study demonstrates how silver may affect the population growth and transmission of *M. congolensis*. Findings from this study will contribute to our understanding of monogenean population dynamics under varying environmental conditions and propose potential approaches for controlling monogenean infections in aquaculture.

## Methods and materials

### Characterization of silver-engineered nanomaterials

The nAg powder that was used to make the stock solution was purchased from Inqaba Biotechnical Industries (Pty) Ltd (Glentham Life Sciences Ltd, product code: GX0585, MW: 107.87, Average particle size: ± 35 nm, UK). The concentration, size distribution, surface charge (zeta potential), agglomeration pattern, and dissolution of the silver nanoparticles were assessed following the protocols described by Klaine et al. (2008), Stone et al. (2010), and Von der Kammer et al. (2012). A stock solution was prepared using Milli-Q water, and working solutions were made by diluting the stock solution in oxygenated aged tap water. The hydrodynamic size distribution and zeta potential of the nAg were measured using dynamic light scattering (DLS) with a Malvern Zetasizer Nano series (NanoZS). The dimensions, morphology, and elemental composition of the nAg were validated with scanning electron microscopy (SEM) (Brno, Czech Republic) paired with an X-Max50 energy-dispersive X-ray spectrometer (EDS) (Oxford Instruments, Halifax, England) and analysed using Aztec 2.1 software (Oxford Instruments, Halifax, England). The nAg solution was sonicated for an hour, and a drop was placed onto a glass slide, which was then dried in a Sanplatec Corp Sanpla dry keeper desiccator cabinet (Kita-Ku, Osaka, Japan). The sample was sputter-coated with carbon (Quorum Q300T ES carbon coater, East Sussex, England) and observed at 20 kV. Furthermore, the size of the primary nAg particles was confirmed using transmission electron microscopy (TEM: FEI Tecnai, G2). For TEM analysis, a drop of the dispersion solution was placed on a carbon-coated copper grid (Protea Laboratory Solutions (Pty) Ltd, product code: S-3630C-MB, Midrand, South Africa), air-dried, and then analysed at high voltage (200 kV). Dissolution of the silver nanoparticles was measured using dialysis tubing strips (product code: D9777, Sigma-Aldrich Ltd, Dorset, UK) with a cellulose membrane (12,000 Da molecular weight). Dissolution was monitored over periods of 0 h, 6 h, 12 h, 24 h, 120 h, and 240 h.

### Reproduction of parasites off the host

**Fig. 1:**
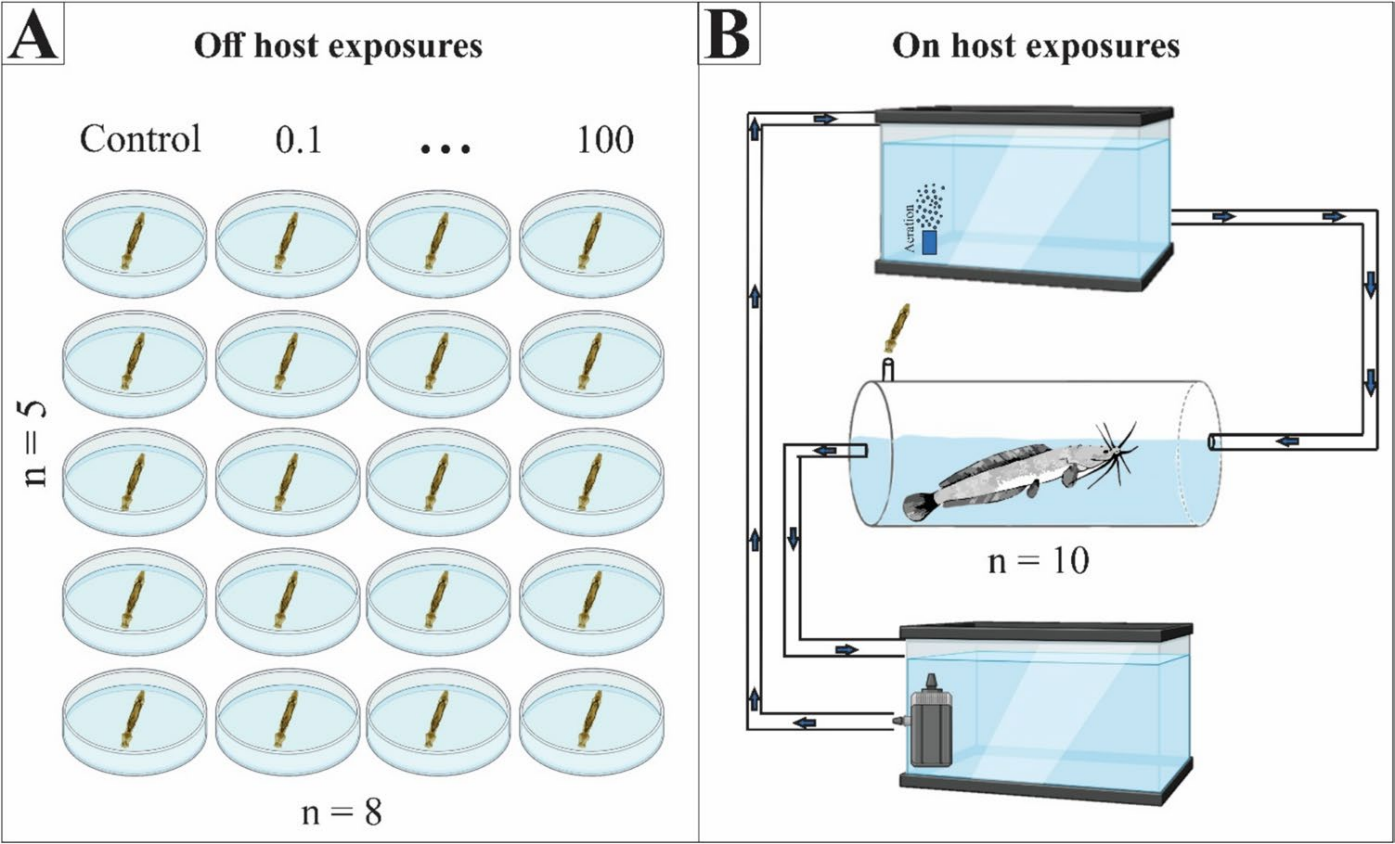
**A** The experimental set-up for observation of the reproduction of parasites (*Macrogyrodactylus congolensis)* off the host, with one specimen per Petri dish. **B** Schematic drawing of the holding facility for observation of reproduction of *Macrogyrodactylus congolensis* on *Clarias gariepinus*. Aerated water flows via a tube from the top reservoir into the tube tank (with gravitation) and exits the tube tank into the bottom reservoir with gravity. The water is pumped from the bottom reservoir to the top reservoir via a compression pump. The fish were placed in the tube tank with sufficient space to turn around and with an air pocket at the top to allow air breathing. Fish were fed through the siphon inserted on top of the tube

Observations of the parasite *M. congolensis* were made on the skin of *C. gariepinus.* A parasite culture was maintained within the UJ Aquarium and kept on *C. gariepinus* in borehole water at 22–23 °C, pH (5.9–7.8) and oxygen (4–7 mg/L). Parasites were collected with a glass slide to scrape the parasites from the surface of the fish. Parasites were transferred to Petri dishes containing aged tap water and immediately exposed. For AgNO_3,_ a concentration range of 0.1 µg/L, 1 µg/L, 2 µg/L, 5 µg/L, 10 µg/L, 20 µg/L, 50 µg/L, and 100 µg/L was used, along with a control with only aged tap water. The concentration range used for nAg was 0.1 mg/L, 1 mg/L, 2 mg/L, 5 mg/L, 10 mg/L, 20 mg/L, 50 mg/L, and 100 mg/L plus a control with only aged tap water. Five parasites were exposed per concentration with only one specimen per Petri dish to record reproduction (Fig. 1A**)**. A 16 h day and 8 h night cycle was maintained throughout the exposure. Parasite mortality was recorded every 30 min for the first 6 h and every 2 h thereafter. The observation concluded with the mortality of the parasites in the control. Mortality was confirmed when no movement occurred and when parasites failed to respond to a gentle touch with a fine paintbrush.

### Reproduction of parasites attached to the host

All fish used in the current study were between 1–2 years old (with an average weight of 0.24 kg and an average total length of 27.1 cm), hatchery-reared African sharptooth catfish (*C. gariepinus)*, purchased from the Free State Department of Agriculture and Rural Development Agriculture Technology Demonstration Centre (ATDC) Fish Hatchery in Gariep Dam, Free State (South Africa). A permit to import live fish (**CPE4-002257**) and a permit to place and release fish into the aquarium at the University of Johannesburg (**CPE3-000386**) were obtained from the Gauteng Department of Agriculture and Rural Development. All procedures involving animals were carried out within guidelines by the South African National Animals Ethics Council following approval by the University of Johannesburg Ethics Committee (**Reference Number: 2023–07–12/Latief_Oldewage**). For the exposures, fish were separated into males and females and individually placed into Perspex tubes (length of 40 cm and diameter of 13 cm) in a closed system (Fig. 1B) with a water flow rate of ± 726 mL per minute, and each fish was individually infected with one parasite by releasing a parasite into the water directly above the fish. Parasites were allowed to attach to their hosts and acclimate for an hour before the start of exposures. The reproduction of the parasites on the fish was observed by physically counting the parasites on each fish every 6 h and recorded for 10 days. The fish parasite system was exposed to the LC_10_ concentrations for AgNO_3_ (0.14 µg/L) and the LC_10_ concentrations for nAg (248.11 µg/L) of the parasite using the same nAg characterized in Latief et al. (2025) to minimise harm to the host fish while still applying concentrations sufficient to elicit measurable effects on the parasite. For each exposure, 10 replicates (five male and five female fish) were conducted. The fish and parasites were kept in tube-shaped tanks with sufficient space to turn around, with an air pocket to allow for the air-breathing required by African sharptooth catfish (Maina 2018) and the tubes to allow for observation and counting of parasites from all angles. Water was circulated to introduce additional compressed air and to emulate a slow water stream flowing over a swimming fish. No parasites were observed in other portions of the flow-through setup throughout the duration of the exposure.

### Spatial distribution of *Macrogyrodactylus congolensis* on host

**Fig. 2:**
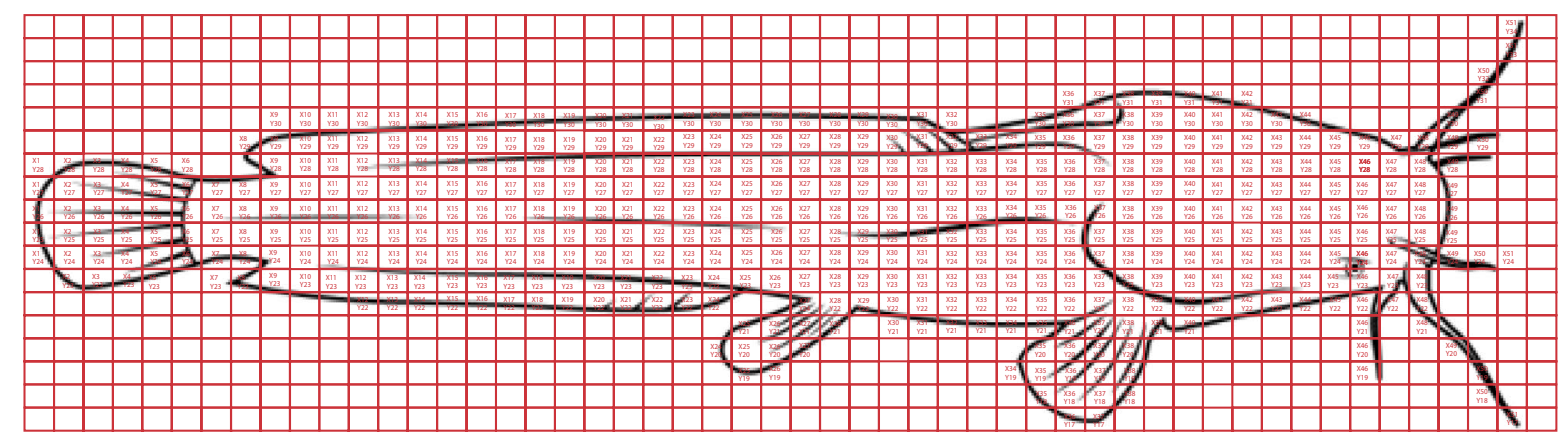
Schematic drawing to illustrate the grid used to construct a heatmap on Heatmapper software of *Macrogyrodactylus congolensis* infecting *Clarias gariepinus*

The reproduction and cumulative spatial distribution of parasites on their host were done simultaneously; a single parasite was observed on an individual host for 10 days, with the parasite numbers and locations recorded manually. To standardise the data reporting, a two-dimensional grid representation of the host was created using CorelDRAW (Fig. 2**)**. The presence of a parasite was recorded within each grid cell. In some cases, multiple parasites occupy the same cell. An identical copy of the image and grid was used for all hosts to ensure a standardised field of view with corresponding grid cells. Using ‘Heatmapper’ (a free online software), a heatmap was generated to visualise parasite distribution on its host. The heatmap colour scale represented parasite density, with red indicating higher concentrations and blue indicating lower concentrations. For the parameters of the pixel data, ChatGPT version o3-mini-high was used. Signals associated with parasites were identified using a threshold of 150. Pixels with values higher than this threshold indicated the presence of parasites. All images were acquired under standardised settings to ensure that every pixel represented a comparable area (± 1.2 µm^2^ per pixel). This enabled direct comparisons among different time points, sexes, and exposures. To eliminate isolated artefacts and reduce background noise, a 3 × 3 median filter (‘Median’ command in ImageJ with a radius of 1 pixel) was used to suppress ‘salt and pepper’ noise. To prevent false positives, clusters with fewer than five consecutive pixels above the threshold were not included. To ensure uniformity in intensity values between treatment groups and time points, brightness and contrast were standardised for every image. A quantitative indicator of parasite burden was created by adding the number of pixels in each anatomical location that exceeded the threshold. After that, these pixel counts were compared between time points (1 h, 120 h, and 240 h) and between sexes (males and females).

### Parasite and host tegument observation with SEM and EDS analysis

The tegument of the parasites and hosts was studied after exposure to 5 µg/L AgNO_3_ and 5 mg/L nAg (*n* = 7). For the control and exposed, fish skin tissue of 1 × 1 cm^2^ × 0.5 mm was cut from the side of the fish at the mid-body, directly over the lateral line, were placed in 70% ethanol, for 6 months, were dehydrated through a graded ethanol series (starting at 80%, 90%, 96%, 96%, 100%, 100%) at 7-min intervals. The samples were then placed into increasing concentrations of hexamethyldisilazane in 100% ethanol (HMDS) (Merck KGaA, Darmstadt, Germany) (40%, 70%, 100%, 100%) at 5-min intervals (Nation 1983; Dos Santos & Avenant-Oldewage 2015) and dried overnight in a Sanpla dry keeper desiccator cabinet (Kita-Ku, Osaka, Japan). Dehydrated specimens were sputter-coated with gold using an Emscope SC500 (Quorum Technologies, Lewes, U.K.) and examined using a Tescan Vega 3 scanning electron microscope (SEM) (Brno, Czech Republic) equipped with an X-Max50 energy-dispersive X-ray spectrometer (EDS) (Oxford Instruments, Halifax, England), operated by Aztec 2.1 software (Oxford Instruments, Halifax, England) for Windows. Elements were expressed in weight percentage (wt %).

### Statistical analysis

Excel and SPSS version 28 for Windows were used to perform the statistical analysis. The normality of the data was assessed using the Shapiro–Wilk test and histograms. Following analysis of normality and equality of means, a one-way analysis of variance (ANOVA) with a post hoc Tukey test was performed to test for statistically significant differences between reproduction in parasites exposed to AgNO_3_, nAg and a control. GraphPad Prism software (Prism 5 for Windows; Version 5.02, California USA) was used to construct a graphical representation.

## Results

### Characterization of nAg

**Fig. 3:**
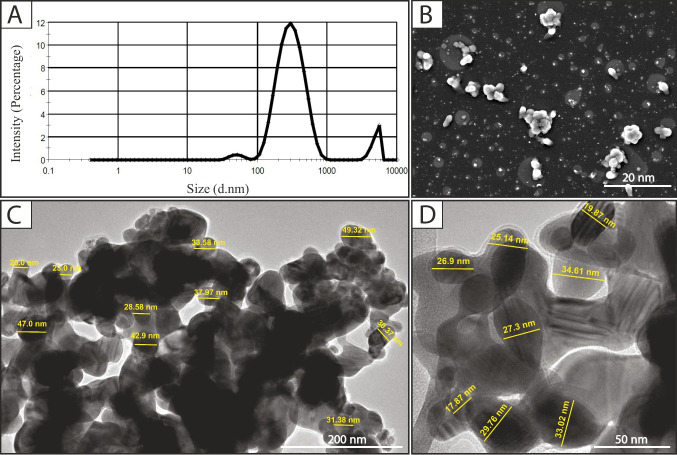
Characterization of nAg. **A** Graph showing the hydrodynamic particle size distribution. **B** Scanning electron micrograph of nAg at 20 nm. **C** and **D** Transmission electron micrograph of nAg showing measurements of the primary particles and agglomeration

The average primary particle size of nAg was 40.3 ± 3.04 nm, but agglomeration occurred (Fig. 3). The average hydrodynamic size distribution was 290.4 nm, and a zeta potential of −18.7 ± 4.69 mV. Minimal dissolution occurred. At low and medium concentrations, dissolution remained consistent over 10 days. However, a dissolution spike occurred between 6 and 24 h at high concentrations.

### Reproduction of parasites off the host

**Fig. 4:**
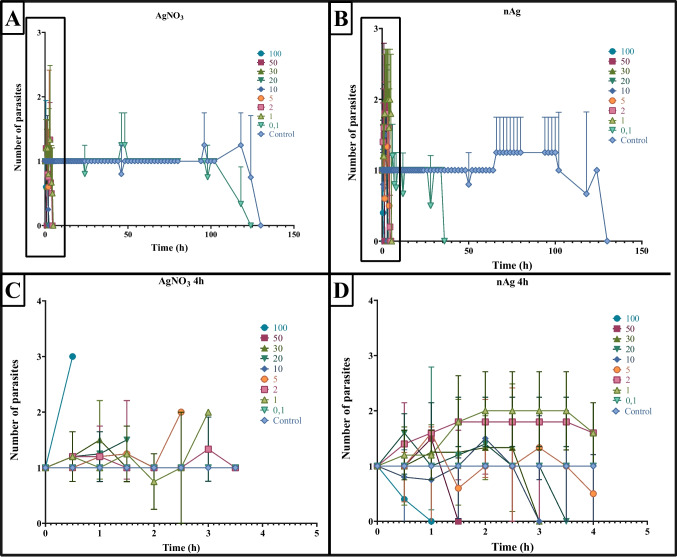
The impact of AgNO_3_ and nAg on the reproductive rate of *Macrogyrodactylus congolensis* off the host at different concentrations. Graphs **A** and **B** show reproduction over 130 h, while graphs **C** and **D** show an expanded view of reproduction over the first 4 h. Error bars represent the standard error for each time point

Reproduction of *M. congolensis* off the host was evaluated under varying concentrations of AgNO_3_ (Fig. 4A and C) and nAg (Fig. 4B and D). Across all treatments, control parasites did not reproduce up until 66 h without the presence of the host. At the low concentrations of silver (0.1–1 µg/L for AgNO_3_ and 0.1–1 mg/L for nAg), reproduction occurred as early as 30 min after exposure for both silvers. Conversely, at the high concentrations (≥ 50 µg/L AgNO₃ and ≥ 50 mg/L nAg), a sharp increase in reproduction was observed shortly after exposure. Notably, in some cases, juveniles were observed at the bottom of the dish, immobile or dead. This indicates that high concentrations of silver may act as a stressor, triggering premature birth before parasite mortality. Overall, these findings suggest that while *M. congolensis* can initiate reproduction off the host under specific stress conditions, but reproductive success is strongly dependent on host presence.

### Reproduction of parasites on the host

**Fig. 5:**
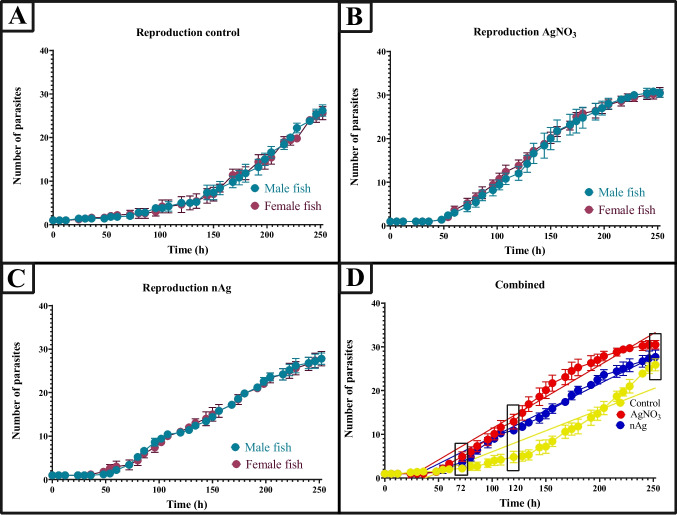
The effect of AgNO_3_ and nAg on the reproduction rate of *Macrogyrodactylus congolensis* kept on *Clarias gariepinus* (males and females presented separately), at the LC_10_ concentration of the parasite. **A** Parasite reproduction in the control group (no silver exposure), **B** parasite reproduction when exposed to ionic silver (Ag), **C** parasite reproduction when exposed to silver nanoparticles (nAg) and **D** a comparison of parasite reproduction patterns across all three conditions (control, Ag, and nAg exposure) after combining data from male and female hosts. Rectangles represent significant differences, and solid lines show the linear regression of each curve. Error bars represent the standard error for each time point

In the control group, the number of parasites increased consistently throughout the study period, with no significant differences between those parasites on male or female fish, suggesting stable and uninhibited reproduction (Fig. 5**)**. In contrast, parasites exposed to AgNO_3_ showed a significant and exponential increase in population from 50 to 200 h, after which their reproductive rate declined. Parasites exposed to nAg displayed a similar growth pattern to the AgNO_3_ group, with an exponential increase in population, higher than that of the control but slightly lower than that observed in the AgNO_3_ group, which eventually reached a plateau. No significant difference was observed between the increase in population size of the parasites on male and female fish therefore, the results were combined to compare the effect of the treatment (*p* = 0.926) (Fig. 5D). When comparing all three groups (control *r*^2^ = 0.86, exposure to AgNO_3_ *r*^2^ = 0.96 and nAg *r*^2^ = 0.97), reproduction between groups showed a statistically significant difference (ANOVA, F = 7.897, df = 2, *p* < 0.001). The results of the multiple comparison Tukey test also showed a statistically significant difference between the control group and AgNO_3_ (*p* < 0.001) and significant differences between the control and nAg groups (*p* = 0.049). For AgNO_3_ and the control, the first significant difference was observed at 36 h (*p* = 0.036). In contrast, at 72 h the first significant difference is seen between the control and nAg (*p* = 0.017). At 120 h, a significant difference was observed between all three groups (control and AgNO_3_: *p* = 0.0167, control and nAg: *p* < 0.0001, AgNO_3_ and nAg: *p* = 0.0209). At 252 h, there is a significant difference only between the control and AgNO_3_ groups (*p* = 0.0002) and AgNO_3_ and nAg (*p* = 0.0004). Compared to the control, which had an exponential increase, the exposed groups had a significant stimulation in reproduction from 72 h, whereas at 252 h, the control showed continuous growth with no plateau, contrary to what was observed in the exposed groups. The difference in reproduction rates between the control and exposed groups suggests that both AgNO_3_ and nAg at LC_10_ concentrations have a moderate but noticeable inhibitory effect on the reproduction of *M. congolensis* after 252 h.

### Spatial distribution of *Macrogyrodactylus congolensis* on the host

**Fig. 6:**
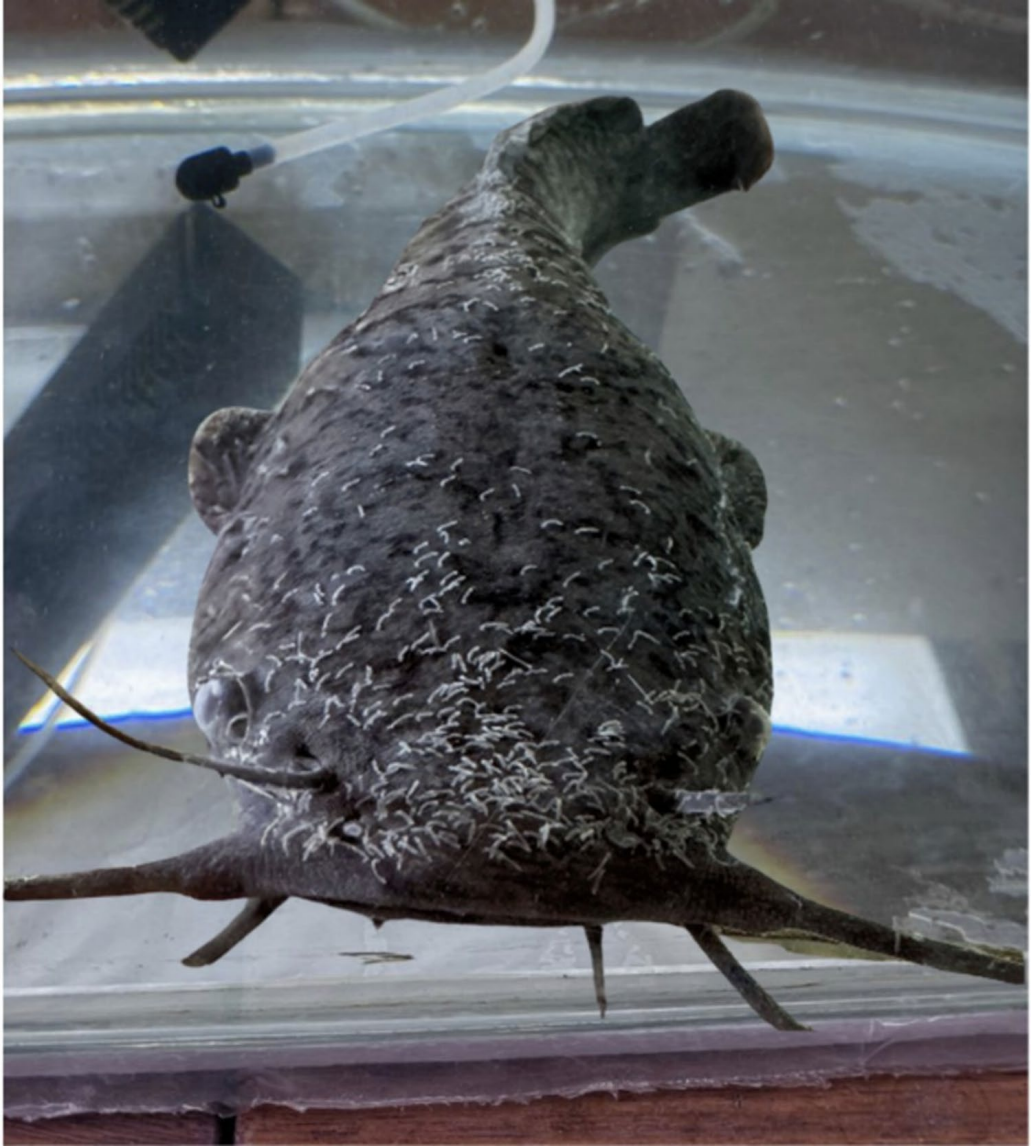
Macrograph of *Clarias gariepinus* with numerous parasites (*Macrogyrodactylus congolensis)* attached to the skin, barbels, fins and even on the eyeballs of the fish

Most parasites are attached to the dorsal side of the head of the fish (Fig. 6). The spatial distribution of *M. congolensis* showed that although the total parasite numbers may remain similar across timepoints or treatments, the distribution and intensity of infection varied between body regions of the fish host. Within the chamber, fish in control tanks exhibited normal behaviour throughout the exposure duration and maintained a steady pattern of swimming and resting. However, as parasite numbers increased, fish were observed to rub against the tank surface. In the exposed groups, fish had bursts of erratic swimming, which was more evident in the AgNO_3_ group, but eventually, fish became more lethargic. Both exposure groups also displayed frequent rubbing against tank surfaces. Quantifying the distribution across the head, body, and fin regions using pixel counts provided a more sensitive and spatially resolved metric and showed subtle shifts in spatial distribution that are easily missed with parasite counts. Additionally, pixel analysis reduced subjectivity and allowed quantification in low and high-intensity infections.

**Fig. 7:**
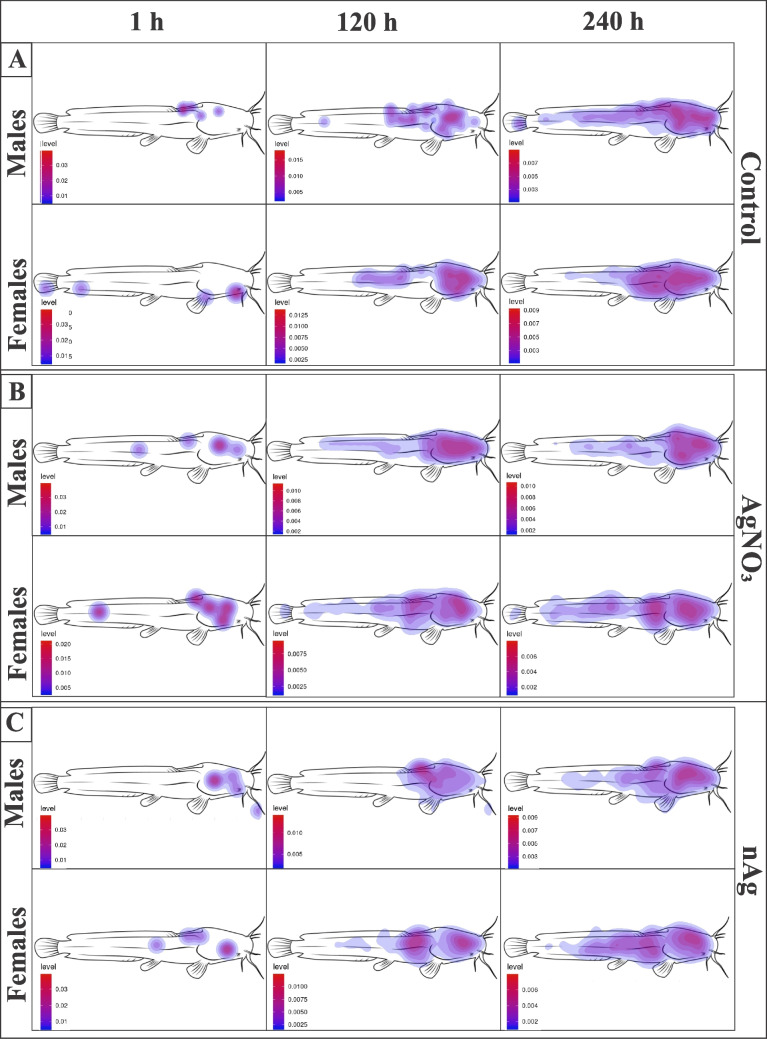
Heatmaps illustrating the spatial distribution of *Macrogyrodactylus congolensis* on its host, *Clarias gariepinus* (males and females), at different time points (1 h, 120 h, and 240 h) for the A: control, B: AgNO_3_, C: and nAg. The study was conducted in control water media (aged tap water) to observe parasite distribution and reproduction over time

The head region consistently showed the highest percentage of pixels (42–53%), regardless of sex, treatment, or timepoint. The body accounted for 25–39% of pixels, while fins showed the lowest (15–27%) (Fig. 7). Results are presented as a percentage of pixels for fish body regions. In the control group for male fish, the dorsal side of the head consistently had the highest number of pixels, accounting for 52.6% of pixels at 1 h, 47.1% at 120 h, and 44.4% at 240 h. The body had 26.3% (1 h), 35.3% (120 h), and 37.0% (240 h), respectively, while the fins had 21.1% (1 h), 17.6% (120 h), and 18.5% (240 h). A similar trend was observed in the control group for female fish, with the dorsal side of the head region having 48.6% of pixels at 1 h, 42.2% at 120 h, and 42.2% at 240 h. The body had 24.3% (1 h), 35.6% (120 h), and 38.2% (240 h), respectively, while the fins had 20.7% (1 h), 18.3% (120 h), and 19.2% (240 h).

In the AgNO_3_ group for male fish, the dorsal side of the head region had 51.7% at 1 h, decreasing to 42.3% at 120 h and 42.6% at 240 h pixel percentages. The body had 27.6% (1 h), 39.4% (120 h), and 38.3% (240 h), respectively, while the fins had 20.7% (1 h), 18.3% (120 h), and 19.2% (240 h). Female fish had pixel percentages of 50.8% (1 h), 40.3% (120 h), and 42.0% (240 h) for the head region. The body had 27% (1 h), 38.9% (120 h), and 39% (240 h), respectively, while the fins had 22.2% (1 h), 20.8% (120 h), and 19% (240 h).

Under nAg exposure, both male and female fish exhibited a consistent trend with the dorsal side of the head region having 54.2% at 1 h, decreasing to 44.3% at 120 h and 42.4% at 240 h pixel percentages for male fish. For male fish, the body had 28.8% (1 h), 38.6% (120 h), and 40.4% (240 h), respectively, while the fins had 16.9% (1 h), 17.1% (120 h), and 17.2% (240 h). Female fish had pixel percentages of 55.7% (1 h), 42.7% (120 h), and 42.2% (240 h) for the head region. The body had 26.2% (1 h), 38.7% (120 h), and 40.2% (240 h), respectively, while the fins had 18% (1 h), 18.7% (120 h), and 17.7% (240 h).

### Observation of parasite tegument with SEM with EDS

**Fig. 8:**
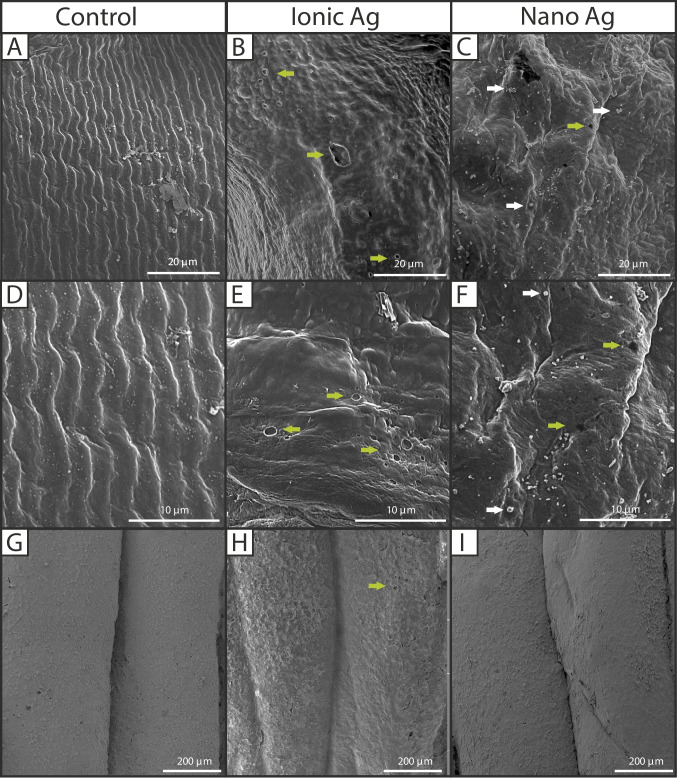
Scanning electron micrographs illustrating the parasite *Macrogyrodactylus congolensis* and fish *Clarias gariepinus* skin under different conditions: **A** and **D** tegument of the control parasite, showing normal surface structure; **B** and **E** parasite tegument following exposure to AgNO_3_; **C** and **F** parasite tegument after exposure to nAg. **G** skin of control fish, **H** fish skin exposed to AgNO_3_ and **I** fish skin exposed to nAg. Green arrows indicate tegument damage to the surface (punctures), and white arrows indicate nAg particles on the tegument

The tegument of *M. congolensis* exposed to AgNO_3_ and nAg showed substantial changes compared to those in the control group (Fig. 8). The tegument of control parasites (Fig. 8A and D) had a well-organised, striated surface with no apparent disruptions or punctures, indicating an intact and healthy tegument. However, specimens exposed to AgNO_3_ (Fig. 8B and E) for 10 days showed punctures, loss of corrugations, formation of vacuoles, punctures and shrivelling, indicating disruption/degradation of the tegument. Similarly, the tegument of parasites exposed to nAg (Fig. 8C and F) showed visible alterations, including punctures, loss of corrugations and the presence of nAg particles on the surface of the tegument. However, tegument damage was visibly less severe than that seen in parasites exposed to AgNO_3_. The SEM/EDS element analysis of *M. congolensis* (Table 1) confirmed compositional differences between particles on the tegument of parasites in the control and parasites that were exposed to either AgNO_3_ or nAg. Silver was absent on the surface of the tegument of parasites in the control group; there was an increase of about 1.3 wt% in Ag on parasites exposed to AgNO_3,_ while those exposed to nAg increased to about 19.8 wt% in nAg aggregates and agglomerates, representing the highest Ag weight percentage among all groups. There was minimal change observed in the weight percentages of other elements between the control and exposed groups. The skin of *C. gariepinus* in the control group (Fig. 8G) showed a smooth epidermal surface with no evidence of surface changes. However, fish exposed to AgNO_3_ (Fig. 8H) showed evidence of localised punctures and a rough skin surface, which could be due to excess mucus formation. In contrast, fish exposed to nAg retained an intact surface with no damage to the skin.

**Table 1:**
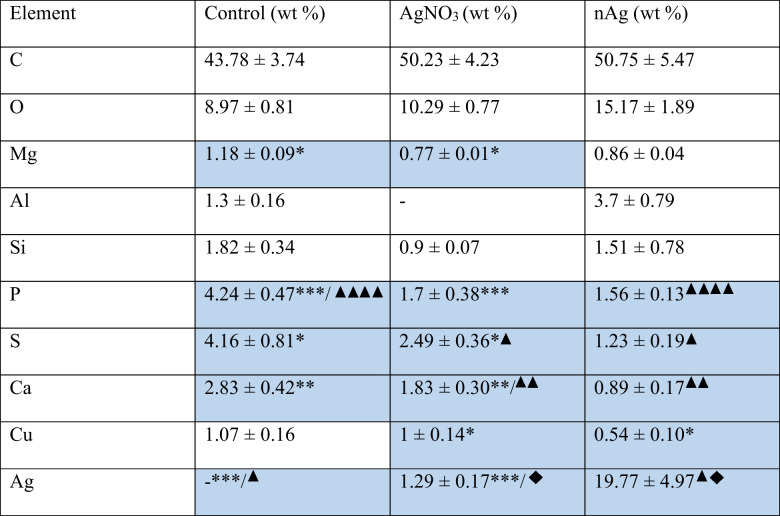
Mean weight percentages for elements detected on *Macrogyrodactylus congolensis* for the control and parasites treated with AgNO_3_ and nAg by SEM/EDS analysis. Results are presented as a mean value ± standard error of the mean

Common symbols and highlighted blocks represent significant differences between groups. The number of symbols corresponds to the level of significance.

## Discussion

For viviparous species like *M. congolensis*, control measures in aquaculture should focus on inhibiting the reproductive cycle to limit population growth (Kearn 1994; Bakke et al. 2007). The current study demonstrates that both AgNO_3_ and nAg present concentration-dependent effects. In trying to find a compromise between host health and parasite toxicity low concentrations of AgNO_3_ (0.1 µg/L to 2 µg/L) and nAg (0.1 mg/L and 1 mg/L) in the current study had a minimal impact on parasite reproduction, compared to those in control conditions, suggesting that low concentrations may not be effective against *M. congolensis* and may have a limited biological impact, consistent with Sures (2001), who reported that sub-lethal concentrations of metals have minimal to no effect on parasite viability.

In off-host trials, at low concentrations, parasites started reproducing at 30 min, suggesting that even minimal silver presence can cause stress to parasites and potentially compromise early development, as juveniles in the AgNO_3_ group died quickly after, whereas juvenile parasites in the nAg group only started to die after a few hours of exposure. The medium concentrations initially stimulated the reproduction of the parasite before inhibiting reproduction, at higher concentrations (50 µg/L for AgNO_3_ and 50 mg/L for nAg), reproduction occurred, but juveniles died soon after birth, or during birth, which led to a rapid decrease in the number of parasites, and the highest concentration (100 µg/L for AgNO_3_ and 100 mg/L for nAg) caused a more substantial inhibitory effect on reproduction, including mortality of adults and juveniles. The initial stimulation in reproductive output is most likely a stress response, and the cessation of reproduction in high concentrations (100 µg/L for AgNO_3_ and 100 mg/L for nAg) were similar to the effects reported in other stress response studies (Marcogliese et al. 1998; Moles & Wade 2001; Khan & Payne 2004) who report that some parasites have an increase in reproduction under moderate stress but a decline in population under severe or continuous stress conditions. The overall decreased number of parasites in medium and high concentrations of silver indicated that effective control of *M. congolensis* requires concentrations (20 µg/L to 100 µg/L for AgNO_3_ and 20 mg/L to 100 mg/L for nAg) high enough to suppress parasite viability. These concentrations coincided with the ultrastructural alterations observed under SEM, including tegument punctures, wrinkling, and particle attachment. This suggests that the decrease in parasite reproduction and survival is closely linked to structural disruption of the parasite tegument. However, the LC_10_ concentrations used in on host exposures of AgNO_3_ and nAg provided moderate inhibition of *M. congolensis* reproduction. Within the first three days, no significant effects were observed, but thereafter, the exposed groups had a stimulation in parasite reproduction, highlighting that exposure times are complex and require long-term studies. Results from the on and off host exposure during the current study, therefore, demonstrate that the host is required for reproductive studies using *M. congolensis*, where a maximum number of parasites observed across the exposure durations was 3. Future studies might consider testing longer continuous exposure times to assess whether silver can fully suppress parasite reproduction due to overexertion or significantly shorter exposure durations at higher concentrations.

This study coincides with the results of previous research indicating that silver-based compounds are effective antiparasitic agents, though their effects vary depending on concentration, exposure duration, and parasite species (Saleh et al. 2017; Pimentel-Acosta et al. 2019). For example, Pimentel-Acosta et al. (2019) used nAg of ~ 35 nm (ARGOVIT) and ~ 1–3 nm (UTSA) against *Cichlidogyrus* spp., which resulted in a concentration-dependent effect. The UTSA caused 100% mortality of adults and eggs at 36 µg/L within 1 h, while ARGOVIT required higher doses (60 µg/L). Rehman et al. (2019) used ~ 8 nm nAg against *Gigantocotyle explanatum* and showed dose-dependent effects at 5–50 µg/mL, including ROS, DNA fragmentation, and severe tegument damage. A study by dos Santos et al. (2024) used nAg of 6.24 ± 2.78 nm (hydrodynamic size 28.37 nm) against *Dactylogyrus minutus*, at concentrations ranging from 100 to 800 mg/L. At high concentrations of 800 mg/L, only 87% of parasites were eliminated. While these studies report effects at lower concentrations than observed in the current study, this could be due to differences in particle size, agglomeration, surface coating and species susceptibility. Although both AgNO_3_ and nAg at LC_10_ concentrations displayed a similar pattern in reproductive output of *M. congolensis*, AgNO_3_ had a faster and more significant increase in parasite numbers over time.

Similarly, the effects on the tegument exposed to both AgNO_3_ and nAg were found to disrupt the tegument integrity, forming major ultrastructural changes, whereas the tegument of the control parasite appears smooth and intact. Parasites exposed to AgNO_3_ in SEM analysis displayed more punctures and shrivelling of the tegument, while exposure to the nAg had minimal punctures, and the presence of nAg particles attached to the tegument. Kar et al. (2014) found that gold nanoparticles caused severe ultrastructural damage, including peeling, punctures and increased permeability to the tegument of *Raillietina* sp., which led to metabolic collapse, starvation, and eventual death of the parasite. Similarly, Abu-Elala et al. (2018) found that chitosan-silver nanocomposites caused severe alterations in the parasite *Lernaea cyprinacea,* which included degeneration of the swimming legs, wrinkles, longitudinal folds and marked swelling in the dermal layer of the parasite and that nanocomposite aggregates on the surface of the eggs. Additionally, Pimentel-Acosta et al. (2019) reported similar findings demonstrating that nAg exposure caused tegmental damage and accumulation of nAg particles on the tegument of *Cichlidogyrus* spp. and demonstrated that nAg also induced DNA damage and deduced that this led to a decreased reproductive output (Pimentel-Acosta et al. 2020).

In the EDS analysis, nAg had the highest weight %; however, this reflects the presence of particles rather than free ions. Significant differences observed for elements other than silver may result from silver binding to sulphur and phosphorus biomolecules, thereby disrupting enzymatic and structural functions and integrity (Jung et al. 2008; Gordon et al. 2010). Silver ions can also interfere with the binding of divalent cations such as Ca^2+^ and Mg^2+^ at membrane or protein binding sites, by competing for negatively charged ligands. This is supported by Schwartz and Playle (2001), who showed that Mg can compete with Ag for binding sites in fish gills and by Wood et al. (1999), who showed that Ag uptake in fish is affected by the concentration of other divalent cations, such as Ca and Mg, in the water. In addition, Ag^+^ can also compete with Cu^+^/Cu2^+^ for binding sites to metalloproteins and redox enzymes (Levard et al. 2012). Therefore, the significant differences observed for Mg, P, S, Ca, and Cu in the current study likely reflect the composition of the parasite's tegument but also the competitive or disruptive interactions of silver with these elements.

The greater effect caused by exposure to AgNO_3_ compared to nAg could be related to its immediate bioavailability and reactivity of Ag^+^ ions, enabling effective interaction with essential biomolecules, leading to rapid cellular damage, oxidative stress, and interference with vital metabolic pathways for wide range of organisms, including bacteria like *Escherichia coli* and *Staphylococcus aureus*, fungi such as *Candida albicans* and *Aspergillus niger*, algae like *Chlamydomonas reinhardtii*, and even higher organisms such as *Macoma balthica*, zebrafish, and mammalian cell lines as described by Marambio-Jones and Hoek (2010). This is caused by ionic Ag interacting with thiol groups in proteins, disrupting cellular functions, increasing permeability and causing cell death in organisms (Jung et al. 2008; Gordon et al. 2010). In contrast, the relatively lower and slower toxicity of nAg is linked to its physicochemical properties. Characterization in the current study revealed that although the average primary particle size of nAg was 40.3 ± 3.04 nm, significant agglomeration occurred in suspension, as indicated by a hydrodynamic size of 255.7 nm. This agglomeration likely reduced the nanoparticles’ surface area and delayed their bioavailability, limiting interactions with biological targets. Furthermore, the zeta potential of −16.7 ± 4.13 mV suggests moderate colloidal stability, which may have contributed to further aggregation and reduced the extent of interaction with negatively charged cell membranes. Minimal dissolution of nAg was observed over 10 days (< 0.21%), and a dissolution spike between 6 and 24 h was observed only at the highest concentration. As a result, the slower and more sustained release of Ag⁺ from nAg leads to a delayed onset of toxic effects. This slow ion release has been shown to reduce acute toxicity (Shobana et al. 2021), although its long-term effects and potential for bioaccumulation in organisms remain a concern (Banu et al. 2021). Supporting this, Shao et al. (2023) showed that ionic silver had immediate effects on microbial organisms compared to the relatively slower activity of nAg, further highlighting the importance of ion‐release, which shows that the rapid Ag^+^ availability increases acute toxicity in AgNO_3_ exposures, while the slower Ag^+^ release from nAg supports its reduced toxicity and delayed impact.

Regarding the spatial distribution of *M. congolensis* on its host in all three groups (control, AgNO_3_ and nAg), parasites initially prefer the dorsal side of the head. Fish infected with ectoparasites are often observed to rub their bodies against surfaces to try and get rid of the parasites (Garcia et al. 2014); for this reason, parasites might prefer the dorsal side of the head to avoid getting scraped off the body of the fish. The observed distribution of *M. congolensis* to the body and fins over time in the control group may be triggered by increased parasite burden to reduce intraspecific competition for resources, while remaining in close proximity to enable access to sexual partners (Rohde 1994). A congener, *Macrogyrodactylus clarii,* predominantly inhabits the gill filaments of the same host (*C. gariepinus*) and even co-infecting host species, suggesting species-specific microhabitat preferences (Arafa et al. 2009). Microhabitat selection was also observed by Twumasi et al. 2022, who conducted a study with one parasite strain per fish from different stocks. The results of their study showed that wild *Gyrodactylus bullatarudis* predominantly infected the head region of its fish host, while wild *Gyrodactylus turnbulli* initially preferred the caudal region but shifted towards the head region of its host over time. In the current study, exposure to Ag increased parasite numbers and thereby increased density as compared to those in the control group. AgNO_3_ had a higher impact on parasite distribution and reproduction, while nano silver had a more subtle effect, but still influenced both the distribution and density of parasites on their host.

In the control group, parasites showed high activity immediately after transfer, showing increased acceleration, distance travelled, and swimming speed within the first hour before slowly decreasing over the next 12 h (Latief et al. 2025). This steady decrease allowed the redistribution seen from the dorsal side of the head to the body of the fish in the heatmaps (parasite px % falls from ~ 52% at 1 h to ~ 44% at 240 h while the body px % rises from ~ 26% to ~ 37%). In contrast, in AgNO_3_ exposures, the behaviour data show no activity for the first 3 h, followed by an increase in acceleration, distance, mobility and swimming speed at 6 h (Latief et al. 2025). The delayed activity matches the shift in pixel percentages from ~ 52% head and 28% body at 1 h to ~ 42% head and 39% body by 120 h. In the nAg group, parasites had a short burst of behaviour changes in the first hour (reflected in 54% on the head and dropping only slightly by 120 h), but all behaviour parameters dropped to near zero by 3 h and remained at baseline thereafter (Latief et al. 2025). This inactivity explains why pixel percentages remain head‐focused (dropping only slightly from ~ 54% at 1 h to ~ 42% at 240 h) with minimal body infections (~ 29% to 40%). Thus, movement could be due to the density as parasite numbers increased from 1 h up to 252 h, parasites moving away from the head to an empty niche on the body, avoiding competition, or a result of reproduction slowing down after the 4th generation at about 252 h because of exposure to AgNO_3_ and nAg.

Although there is an urgent need to control parasite infection in aquaculture facilities, the results of this study suggest that while silver compounds can suppress *M. congolensis* reproduction and cause tegument damage, their practical use as antiparasitic agents in aquaculture remains uncertain. Furthermore, the high cost associated with nanotechnologies, the superior performance of ionic silver in this study, and the scale of aquaculture facilities suggest that further research is needed before upscaling. At low, host-tolerated concentrations, the inhibitory effects are only moderate and insufficient for parasite eradication, whereas higher, more effective concentrations have the risk of toxicity to the fish host and potentially to non-target organisms. The reproduction plateau observed in the current study in the exposed groups at 252 h suggests that silver treatment could be considered as a potential parasite management strategy to control gyrodactylid populations in aquaculture. The study suggests that the LC_10_ levels are too low for therapeutic purposes, that the host tolerates it well, but that it will not completely eradicate *M. congolensis* in high-density aquaculture systems. Higher concentrations, extended exposure durations or repeated treatments of strictly on-host exposures would need to be explored to effectively control parasite populations. This should be done with caution, studying the effect on the host skin, gills and pseudobranchs as not to cause toxicity to non-target organisms, including fish hosts, as silver is known to be toxic to fish at high concentrations, impacting gill and osmoregulatory functions (Wood et al. 1999; Bilberg et al. 2012).

## Data Availability

No datasets were generated or analysed during the current study.
